# Novel cooperative pathway of c-Myc and Furin, a pro-protein convertase, in cell proliferation as a therapeutic target in ovarian cancers

**DOI:** 10.18632/oncotarget.23322

**Published:** 2017-12-15

**Authors:** Junko Hasegawa-Minato, Masafumi Toyoshima, Masumi Ishibashi, Xuewei Zhang, Shogo Shigeta, Carla Grandori, Kazuyuki Kitatani, Nobuo Yaegashi

**Affiliations:** ^1^ Department of Obstetrics and Gynecology, Tohoku University Graduate School of Medicine, Sendai, Japan; ^2^ Tohoku Medical Megabank Organization, Tohoku University Graduate School of Medicine, Sendai, Japan; ^3^ Division of Human Biology, Fred Hutchinson Cancer Research Center, Seattle, WA, USA; ^4^ SEngine Precision Medicine, Seattle, WA, USA

**Keywords:** c-Myc, Furin, ovarian cancer, synthetic lethal, Notch1

## Abstract

c-Myc is a master regulator of various oncogenic functions in many types of human cancers. However, direct c-Myc-targeted therapy has not been successful in the clinic. Here, we explored a novel therapeutic target, which shows synthetic lethality in c-Myc-driven ovarian cancers, and examined the molecular mechanism of the synthetic lethal interaction. By high throughput siRNA screening with a library of 6,550 genes, Furin, a pro-protein convertase, was identified as the top hit gene. Furin inhibition by siRNA or a Furin inhibitor significantly suppressed cell proliferation in high c-Myc-expressing ovarian cancer cells compared with low c-Myc-expressing cells. Conversely, Furin overexpression in the presence of high c-Myc significantly promoted cell proliferation compared with only c-Myc or Furin overexpression. Notch1, one of the Furin substrates, was upregulated by c-Myc, and Notch1 cleaved by Furin increased cell proliferation of high c-Myc-expressing ovarian cancer cells. Notch1 was involved in the cooperative pathway of c-Myc and Furin in cell proliferation. In clinical ovarian cancer specimens, co-expression of c-Myc and Furin correlated with poor survival. In conclusion, we found that c-Myc cooperates with Furin to promote cell proliferation. Furin may be a promising therapeutic target in c-Myc-driven ovarian cancer.

## INTRODUCTION

c-Myc is one of the most commonly altered oncogenes in many types of human cancers. It increases DNA synthesis, transcription, RNA processing, synthesis of ribosomal proteins, and regulates the activities of metabolic pathways. These characteristics provide cells with uncontrolled proliferation and independence of growth factors. [1]. Thus, c-Myc is a master regulator in a variety of oncogenic functions.

Ovarian cancer is the most lethal gynecological cancer, and most patients are diagnosed at advanced and metastatic stages [2]. c-Myc overexpression and gene amplification are observed in ovarian cancers [3, 4], which contributes to cancer progression. According to The Cancer Genome Atlas, c-Myc amplification is observed in 31.49% of ovarian cancers [5] and has the highest frequency in solid cancers [6]. c-Myc amplification does not correlate with poor prognoses of ovarian cancer [4, 7], although it correlates in breast cancer [8], prostate cancer [9], and hepatocellular carcinoma [10]. The collaboration between c-Myc and other molecules may promote cancer progression, and correlate with a poor prognosis.

Some cancer cells depend on specific oncogenic pathways for their survival, so-called oncogene addiction [11]. This phenomenon provides a rationale for molecular targeted therapy, such as the BCR-ABL (breakpoint cluster region protein-Abelson murine leukemia viral oncogene homolog 1) fusion gene in chronic myelogenous leukemia, EGFR (epidermal growth factor receptor) mutations and ALK (anaplastic lymphoma kinase) fusion in non-small cell lung carcinoma [12, 13]. However, c-Myc is a transcription factor without a druggable domain, and it is difficult to make c-Myc itself a therapeutic target [14, 15]. Moreover, because c-Myc is essential for proliferating cells, inhibition of c-Myc functions could lead to unacceptable toxicity. Therefore, synthetic lethality as an alternative approach is required, which is an effective therapeutic strategy for c-Myc-driven human cancers [16–22].

A strategy of synthetic lethality for c-Myc-driven ovarian cancer has not been reported so far. Previous studies have revealed partners of synthetic lethality in ovarian cancer, such as PARP [poly (ADP)-ribose polymerase] against BRCA (breast cancer susceptibility protein)-deficient cells [23] and EZH2 (enhancer of zeste homolog 2) against ARID1A (AT-rich interactive domain-containing protein 1A)-mutated cells [24]. Development of a therapeutic strategy for c-Myc-driven ovarian cancer will greatly advance precision medicine.

In this study, we identified Furin, a calcium-dependent serine protease that belongs to the pro-protein convertase family, as a novel therapeutic target that shows synthetic lethality in c-Myc-driven ovarian cancers by high throughput siRNA screening. Furthermore, we propose a novel cooperative pathway of c-Myc and Furin in promoting cell proliferation of ovarian cancer.

## RESULTS

### Identification of synthetic lethal genes in c-Myc-driven ovarian cancer by high throughput siRNA screening

**Figure 1:**
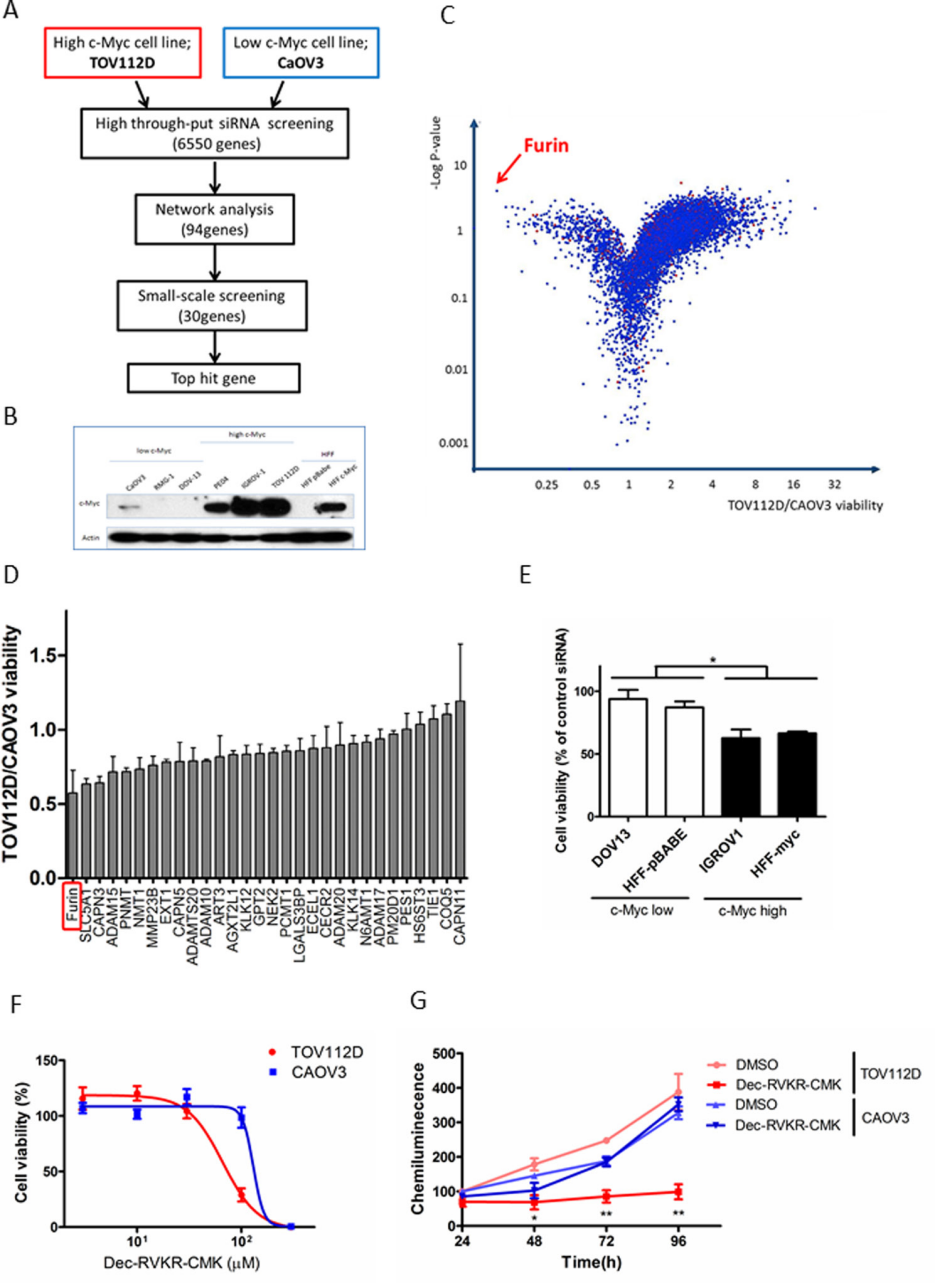
Identification of synthetic lethal genes in c-Myc-driven ovarian cancer by high throughput siRNA screening (**A**) Schematic of siRNA screening. Two ovarian cell lines, TOV112D and CAOV3, were transfected with siRNAs targeting 6,550 genes, and then network analysis was conducted for the top 94 genes. Small scale screening was performed for 30 genes selected from the results of high throughput siRNA screening and network analysis, and the top hit gene was determined. (**B**) c-Myc expression in ovarian cancer cell lines and HFFs. Proteins were subjected to immunoblot analysis with antibodies specific for c-Myc and β-actin. Equal amounts of protein (5 μg) were loaded in each lane. (**C**) Volcano plot of all target genes displaying the relationship between fold change and statistical significance in TOV112D and CAOV3 cell viability. The y-axis is the negative log10 of *P*-values and the x-axis is the ratio of cell viability between two cell lines as measured in log2. Each dot indicates the corresponding gene. The red arrow indicates the top hit gene, Furin, which showed synthetic lethality with c-Myc. (**D**) Validation assay of 30 hit genes was conducted using new siRNA sequences . The y-axis is the TOV112D/CAOV3 cell viability ratio. Values are means ± standard error (SE). (**E**) Cell viability was assessed after Furin siRNA transfection for 72 h in other cell lines: IGROV1 and HFF-c-Myc (high c-Myc), and DOV13 and HFF-p-Babe (low c-Myc). Values are means ± SE. ^*^p < 0.05. (**F**) TOV112D and CAOV3 cells were treated with the indicated concentrations of Dec-RVKR-CMK, a small molecule inhibitor of Furin, for 72 h, and then cell viability was assessed. Values are means ± SE. (**G**) TOV112D and CAOV3 cells were treated with DMSO (control) or 100 μM Dec-RVKR-CMK for the indicated times. Cell proliferation was assessed using CellTiter-Glo. Values are means ± SE. ^*^p < 0.05. ^**^p < 0.01.

We carried out high throughput siRNA screening with a library of 6,550 genes (Figure 1A). Immunoblot analysis revealed protein expression of c-Myc in six ovarian cancer cell lines and human foreskin fibroblasts (HFFs) (Figure 1B). Two ovarian cell lines, TOV112D (high c-Myc) and CaOV3 (low c-Myc), were employed for screening, and their cell viabilities were assessed after siRNA transfection for 72 h. The TOV112D/CAOV3 cell viability ratio and negative log *P*-value of each well for each gene were plotted on a volcano plot (Figure 1C). We focused on genes plotted in the upper left area. The top hit gene that showed synthetic lethality with c-Myc was Furin, a pro-protein convertase. Network analysis of 94 genes selected as hits based on TOV112D/CAOV3 cell viability by IPA (ingenuity pathway analysis) revealed a highly connected protein-protein interaction with components playing a role in protein degradation, such as Furin, a disintegrin and metalloproteinase (ADAM), and calpain (CAPN) (Supplementary Figure 1). We selected 30 genes based on potential involvement in cancer pathways and the results of high throughput siRNA screening and network analysis. To validate these genes, we performed small scale validation screening with newly synthesized siRNA. Furin knockdown resulted in the lowest TOV112D/CAOV3 cell viability with a ratio of 0.57 (Figure 1D). We further examined the effects of Furin using multiple cell lines. Pairs of other high and low c-Myc cell lines, IGROV1 and HFF-c-Myc (high c-Myc), and DOV13 and HFF-p-Babe (low c-Myc), were adopted, and cell viabilities were measured after Furin siRNA transfection. Furin knockdown resulted in decreased cell viability of high c-Myc cell lines compared with that of low c-Myc cell lines (Figure 1E). A similar result was obtained by treating cell lines with Furin inhibitor, Dec-RVKR-chloromethyl ketone (CMK). TOV112D cells were more sensitive to Dec-RVKR-CMK compared with CAOV3 cells. IC_50_ (half maximal inhibitory concentration) values were 66 μM in TOV112D cells and 130 μM in CAOV3 cells (Figure 1F). Furthermore, cell proliferation was measured at the indicated time points after Dec-RVKR-CMK treatment. From 48 to 96 h, cell proliferation was significantly suppressed by 100 μM Dec-RVKR-CMK in TOV112D cells. The percentage of growth inhibition was 65.5% compared with dimethyl sulfoxide (DMSO) treatment at 72 h. However, proliferation was not suppressed in CAOV3 cells (Figure 1G).

Through this large scale functional screening, we identified Furin as a novel potential target that shows a synthetic lethal interaction with c-Myc in c-Myc-driven ovarian cancers, and demonstrated that genetic and pharmacological inhibition of Furin led to growth inhibition of c-Myc-driven ovarian cancer cells.

### Furin inhibition arrests the cell cycle at G1/G0 phase and subsequently causes apoptotic cell death

**Figure 2:**
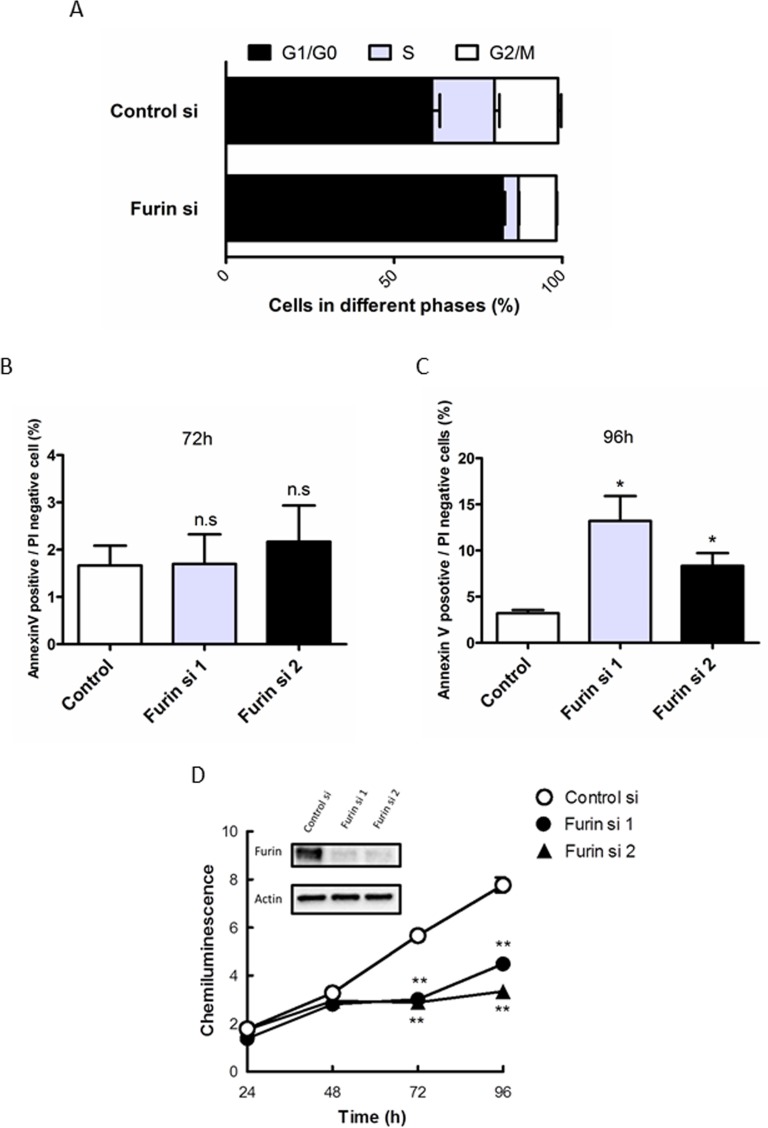
Furin inhibition arrests the cell cycle at G1/G0 phase and subsequently causes apoptotic cell death (**A**) TOV112D cells were transfected with 5 nM control or Furin siRNA #1 for 72 h. After fixation, cells were stained with PI. Cell cycle distribution was determined by flow cytometry. Values are means ± SE. (**B**, **C**) TOV112D cells were transfected with 5 nM control siRNA, or Furin siRNAs #1 or #2. Representative ratios of apoptotic cells positive for annexin V and negative for PI at 72 h (B) and 96 h (C) after transfection are shown. Values are means ± SE. (**D**) TOV112D cells were transfected with 5 nM control siRNA, or Furin siRNAs #1 or #2 for the indicated times. Cell proliferation was assessed using CellTiter-Glo. Furin expression after Furin siRNA #1 or #2 transfections in immunoblot analysis is shown. Values are means ± SE. ^**^p < 0.01.

To determine the mechanism underlying the antiproliferative effects of Furin siRNA, we first investigated the effect on the cell cycle by flow cytometric analysis. Furin knockdown in TOV112D cells increased the percentage of cells in G1/G0 phase from 61.2% to 82.2% and decreased the percentage of cells in S phase from 18.5% to 4.7% at 72 h after transfection (Figure 2A). As a result, Furin inhibition resulted in G1/G0 cell cycle arrest. Next, we investigated the effect on apoptotic cell death using annexin V/propidium iodide (PI) staining. Furin knockdown in TOV112D cells did not change the proportions of annexin V-positive and PI-negative cells compared with control siRNA at 72 h after transfection (Figure 2B). However, at 96 h, Furin knockdown significantly increased the proportions of annexin V-positive and PI negative cells from 3.2% (control siRNA) to 13.2% and 8.3%, respectively (Figure 2C). Furthermore, we assessed proliferation of TOV112D cells at the indicated time points after Furin knockdown. Immunoblotting showed decreased protein expression of Furin. From 72 to 96 h, Furin knockdown significantly suppressed cell proliferation compared with control siRNA (Figure 2D).

These results indicated that cell cycle arrest at G1/G0 and subsequent apoptotic cell death caused the lethality induced by Furin inhibition in c-Myc-driven ovarian cancer.

### c-Myc cooperates with Furin to promote cell proliferation

**Figure 3:**
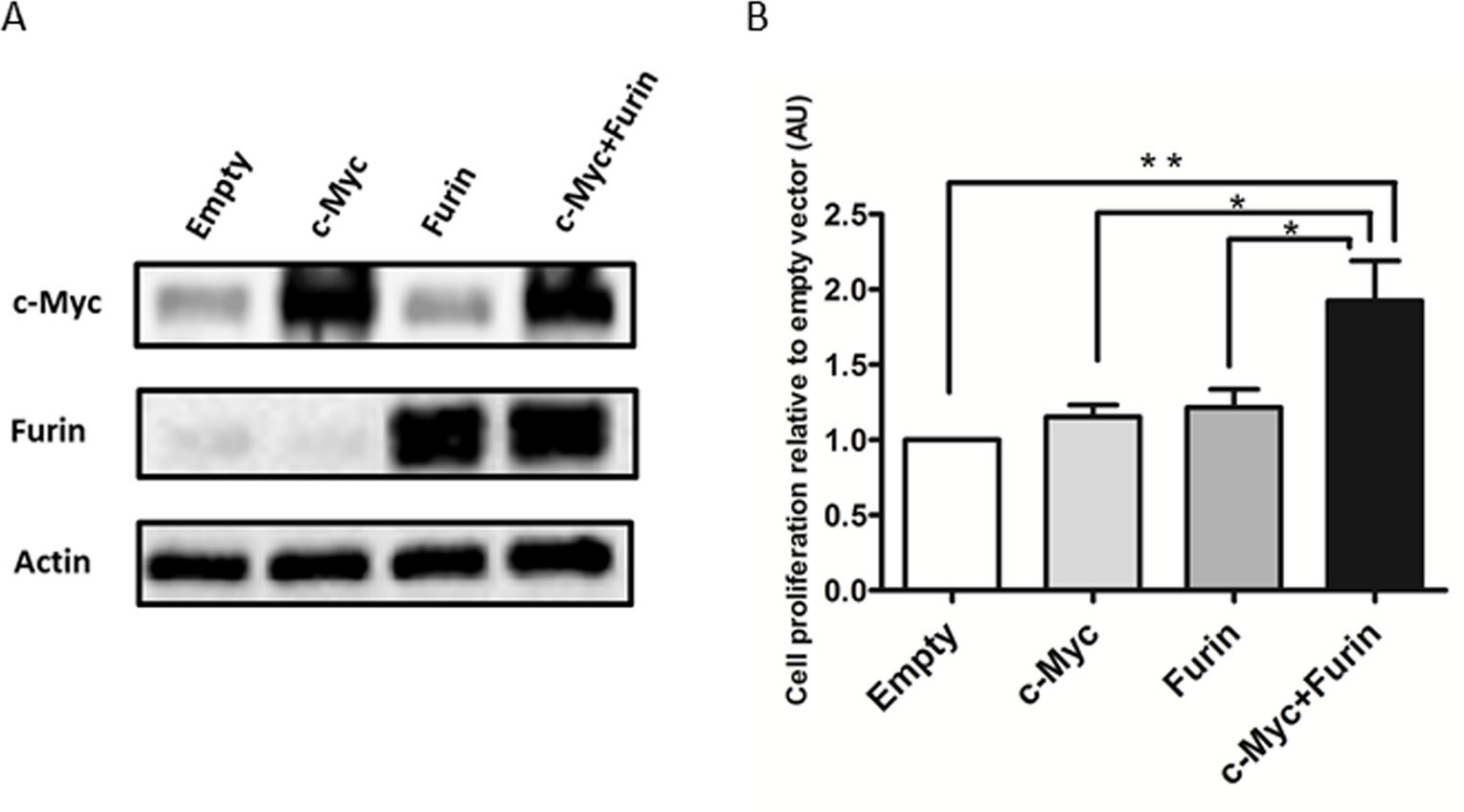
c-Myc cooperates with Furin to promote cell proliferation (**A**) OSE2 cells plated on 3.5-cm dishes were transfected with empty, V5-tagged c-Myc, Furin, or c-Myc and Furin expression vectors (2 μg cDNA) for 24 h before harvesting. Extracted proteins were subjected to immunoblot analysis with antibodies specific for Furin or c-Myc. (**B**) OSE2 cells (3 × 10^3^/well) seeded on 96-well plates were transfected with the indicated vectors (0.1 μg cDNA). Proliferation of OSE2 cells was assessed at 72 h after transfection using CellTiter-Glo. Data shown were normalized to chemiluminescence of the empty vector group. Values are means ± SE. ^*^p < 0.05. ^**^p < 0.01.

To reveal the molecular mechanism of the synthetic lethal interaction between c-Myc and Furin, the effects on overexpression of c-Myc and/or Furin were assessed. The normal ovarian surface epithelium (OSE) 2 cell line, which showed low endogenous c-Myc and Furin expression, was selected and transfected with empty, c-Myc, Furin, or both c-Myc and Furin expression vectors (Figure 3A). Co-overexpression of c-Myc and Furin significantly increased cell proliferation compared with the empty vector at a 1.92-fold, whereas overexpression of c-Myc or Furin didn’t increase (Figure 3B). Similar results were obtained in CAOV3 cells that were employed for screening as a low c-Myc ovarian cancer cell line (Supplementary Figure 2A and 2B).

These results indicated that c-Myc cooperated with Furin to promote cell proliferation, and this collaboration did not act in a c-Myc-Furin axis interaction because overexpression of only c-Myc or Furin did not alter the expression of the other in immunoblot analysis (Figure 3A).

### Upregulation of Notch1 by c-Myc contributes to cell proliferation

**Figure 4:**
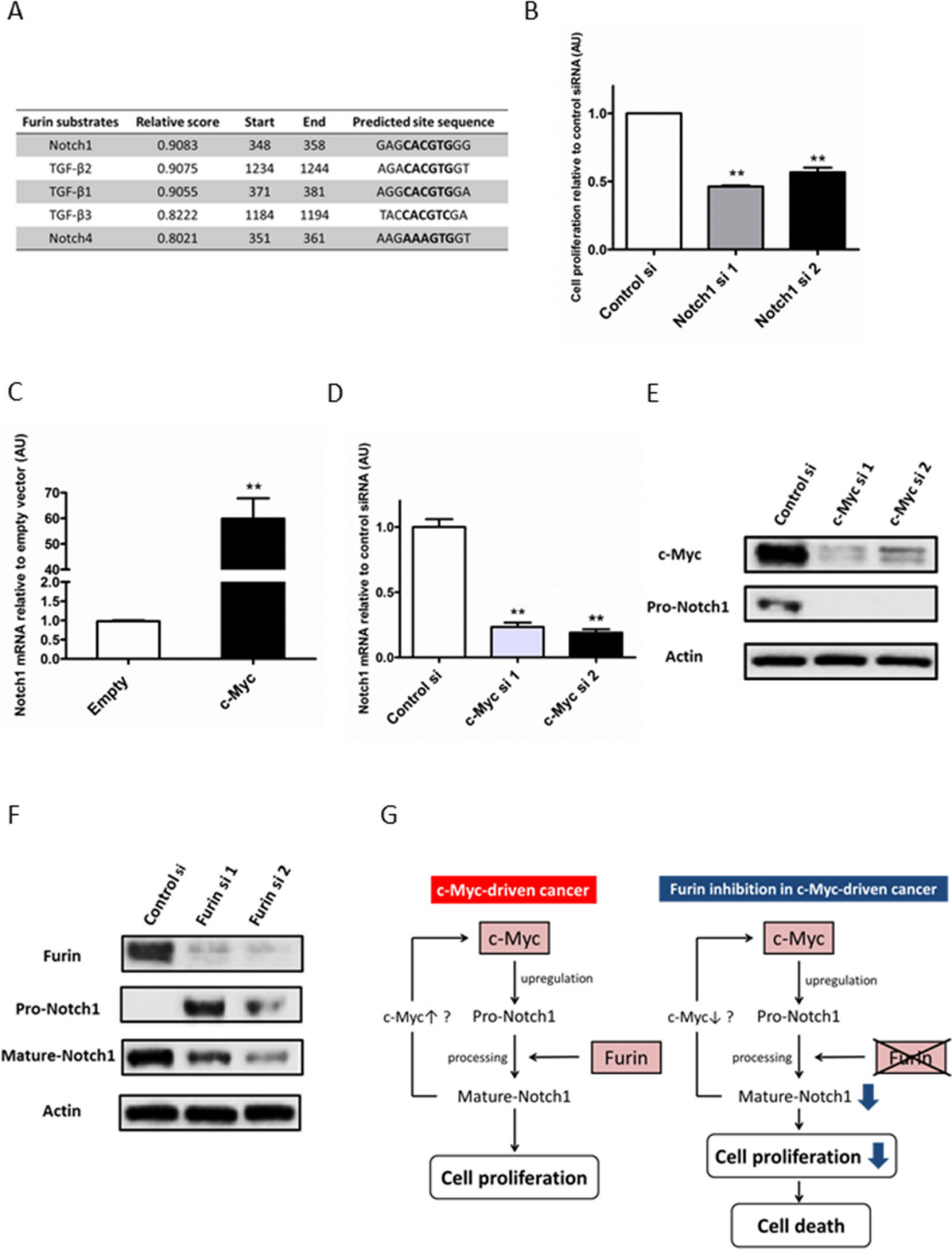
Upregulation of Notch1 by c-Myc contributes to cell proliferation (**A**) Promoter region sequences of Furin substrates were obtained from the NCBI online database. Myc-Max-binding sites in promoter regions were predicted by the JASPAR database. (**B**) TOV112D cells (7 × 10^4^/well) seeded on 6-well plates were transfected with 5 nM control siRNA, or Notch1 siRNAs #1 or #2, and the cell number was measured at 72 h after transfection. Data shown were normalized to the cell number of control siRNA. Values are means ± SE. ^**^p < 0.01. (**C**) TOV112D cells were transfected with 1 μg empty or c-Myc expression vectors for 24 h. Notch1 mRNA expression levels were determined by quantitative real-time PCR. (**D**) TOV112D cells were transfected with 5 nM control siRNA, or c-Myc siRNAs #1 or #2 for 48 h. Notch1 mRNA expression levels were determined by quantitative real-time PCR. Values are means ± SE. ^**^p < 0.01. (**E**) TOV112D cells were transfected with 5 nM control siRNA, or c-Myc siRNA #1 or #2 for 72 h. Notch1 protein expression was analyzed by immunoblotting with antibodies specific for Notch1 and β-actin. Equal amounts of protein (5 μg) were loaded in each lane. (**F**) TOV112D cells were transfected with 5 nM control or Furin siRNAs for 72 h. Notch1 protein expression was analyzed by immunoblotting with antibodies specific for Notch1 and β-actin. The antibody against Notch1 detected the full-length (pro-form) and non-transmembrane region including the Notch1 intracellular domain (mature form) (**G**) Model illustrating the proposed mechanism of the synthetic lethal interaction between c-Myc and Furin.

To investigate the mechanism of the interaction between c-Myc and Furin, we focused on the substrates of Furin. Furin is a pro-protein convertase located in the trans-Golgi network and cell membrane, which is a ubiquitously expressed enzyme in tissues [25]. After Furin cleaves pro-proteins at the carboxyl terminal with basic amino acid sequence RXK/RR [26], these proteins change to a mature form and gain biological activity. Furin cleaves many substrates such as transforming growth factor (TGF)-β, insulin-like growth factor receptor (IGFR)-1, platelet-derived growth factor (PDGF)-A and B, vascular endothelial growth factor (VEGF), membrane type 1-matrix metalloproteinase (MMP), and MMP2. These mature proteins promote cancer cell invasion, metastasis, and proliferation [27–30]. We hypothesized that a substrate of Furin might be a target gene of c-Myc. c-Myc promotes expression of the pro-protein that receives processing by Furin and changes to the mature protein. This processed mature protein then promotes cell proliferation. We particularly focused on growth factors and their receptors, such as TGF-β1–3, insulin-like growth factor (IGF)-1 and 2, IGFR-1, PDGF-A and -B, VEGF-A–C, and Notch1–4, because Furin played an important role in cell proliferation in the presence of c-Myc (Figure 3B). Based on the JASPAR database [31] and previous reports [32, 33], we selected TGF-β1, β2, β3, and Notch1 and 4 as candidates (Figure 4A). Notch4 was excluded because its expression was very low in ovarian cancer cell lines (data not shown).

We first examined whether TGF-β1–3 and Notch1 promoted the proliferation of ovarian cancer cells, because previous reports demonstrated that these substrates have both promotive and inhibitive effects on cancer cell proliferation [34–36]. TGF-β1 significantly increased the cell number at 72 h of treatment, whereas TGF-β3 decreased the cell number, and TGF-β2 did not affect cell proliferation (Supplementary Figure 3). Notch1 inhibition by siRNA significantly decreased the cell number at 72 h after siRNA transfection. Compared with control siRNA, the percentage of growth inhibition by Notch1 siRNAs #1 and #2 was 54.0% and 44.0%, respectively (Figure 4B). These results indirectly indicated that Notch1 promoted cell proliferation. Because TGF-β2 and 3 did not promote cell proliferation, these substrates were ruled out. Next, we examined whether mRNA levels of TGF-β1 and Notch1 were upregulated by c-Myc. The TGF-β1 mRNA level was downregulated by c-Myc overexpression and upregulated by c-Myc knockdown (Supplementary Figure 4A and 4B), whereas the Notch1 mRNA level was upregulated by c-Myc overexpression and downregulated by c-Myc knockdown (Figure 4C and 4D). Moreover, Notch1 and c-Myc mRNA levels correlated in 18 cell lines including 16 ovarian cancer cell lines and HFFs (Pearson’s correlation, *R* = 0.526, Supplementary Figure 5). Therefore, mRNA levels of Notch1 rather than TGF-β1 were upregulated by c-Myc. Notch1 regulation by c-Myc was also found at not only mRNA but also protein levels. (Figure 4E). Taken together, upregulation of Notch1 by c-Myc contributed to cell proliferation in ovarian cancer. Pro-Notch1 is first cleaved by Furin in the trans-Golgi network to produce the biologically mature form [37]. Therefore, we confirmed whether Notch1 received processing by Furin in ovarian cancer cells. Furin inhibition by siRNA increased pro-Notch1 and decreased mature Notch1, indicating the inhibition of Notch1 processing (Figure 4F).

These results suggested that Notch1, one of the Furin substrates, was a key molecule in the cooperative interaction between c-Myc and Furin in cell proliferation (Figure 4G).

### Clinical significance of Furin and c-Myc protein expression in ovarian cancer

**Figure 5:**
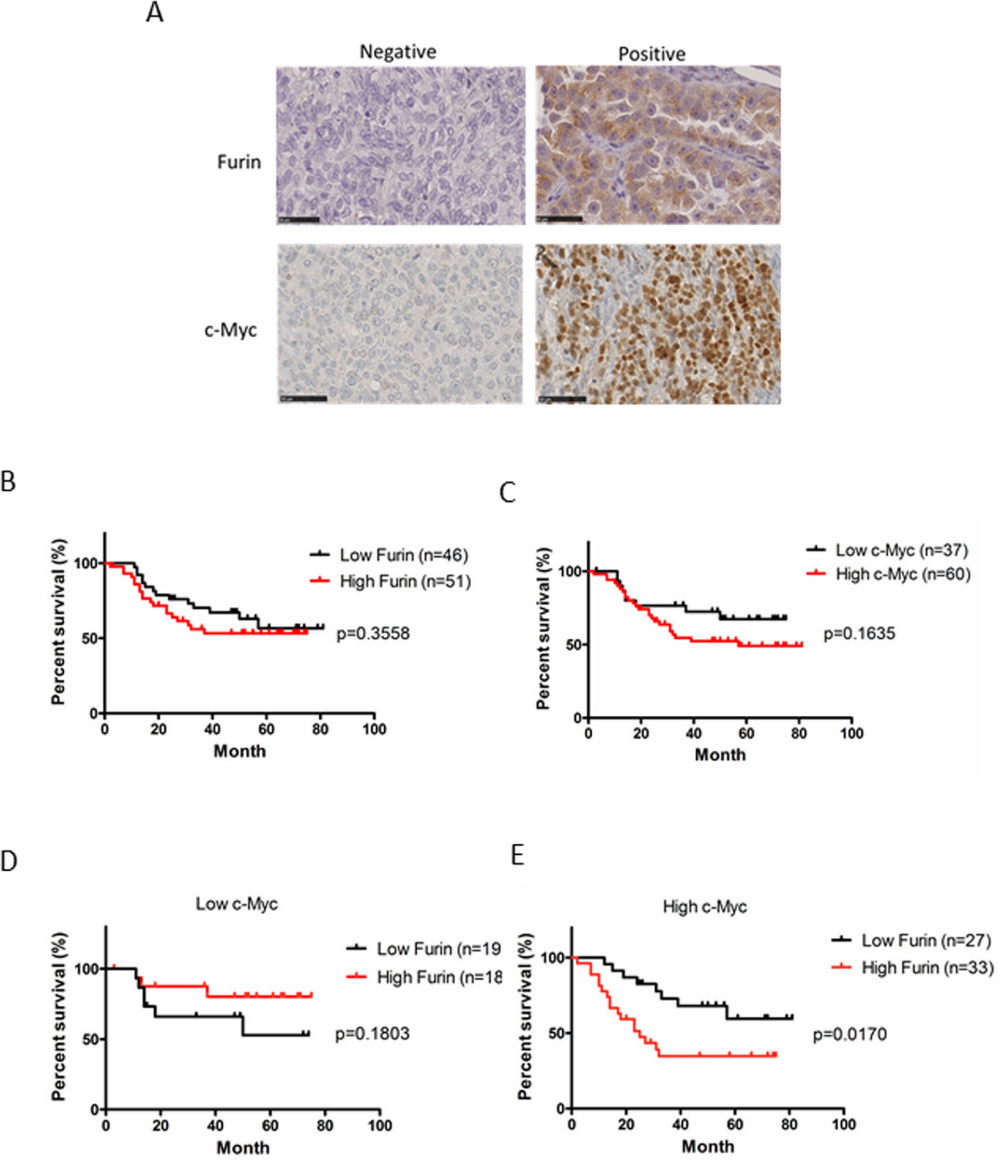
Clinical significance of the protein expression of Furin and c-Myc in ovarian cancer (**A**) Ovarian tumors from patients were subjected to immunohistochemical staining of Furin and c-Myc. Representative examples of positive and negative immunostaining of Furin (upper images) and c-Myc (lower images) in tissues of ovarian tumors are shown (original magnification, ×400). (**B**) Kaplan-Meier curves of disease-free survival (DFS) based on Furin expression. Red indicates high expression and black indicates low expression. (**C**) Kaplan-Meier curves of DFS based on c-Myc expression. Red indicates high expression and black indicates low expression. (**D**, **E**) Kaplan-Meier curves of DFS based on Furin expression in low and high c-Myc groups. Red indicates high Furin expression and black indicates low Furin expression.

To determine the clinical significance of Furin and c-Myc protein expression in ovarian cancer, we analyzed their protein expression patterns in 97 patient specimens of primary tumors by immunohistochemistry and the correlation of Furin and c-Myc expression with patient survival. Furin was stained in the cytoplasm and c-Myc was stained in the nucleus (Figure 5A). Previous reports have shown that Furin expression correlates with poor prognoses of ovarian cancer [38]. Fifty-three percent (*n* = 51) of tumors showed high Furin expression. No significant difference between high and low expression was found in terms of disease-free survival (DFS) (Figure 5B). Sixty-two percent (*n* = 60) of the tumors showed high c-Myc expression. No significant difference between high and low expression was found in terms of DFS (Figure 5C).

To explore the effect of Furin on c-Myc-driven ovarian cancer, we classified the patients into two groups based on c-Myc expression. In the low c-Myc group, 51% (*n* = 19) had low high Furin and 49% (*n* = 18) had high Furin. The Furin expression level did not correlate with survival of patients with low c-Myc expression (Figure 5D). In the high c-Myc group, 45% (*n* = 27) had low Furin and 55% (*n* = 33) had high Furin. Patients with high Furin expression had significantly shorter DFS (*p* = 0.017; Figure 5E) compared with low Furin expression. There was no significant difference between two groups in age, stage and histological type (Table 1). This result suggested that co-expression of c-Myc and Furin correlates with poor prognoses of ovarian cancer though only expression of c-Myc or Furin doesn’t correlate with prognosis.

**Table 1:** Patient characteristics of low and high Furin groups in high c-Myc group (*n* = 60)

	Low Furin (*n* = 27)	High Furin (*n* = 33)	*P*-value
**Age**	52.8	56.3	0.3248
**Stage** (%)			
I	6 (22.2)	9 (27.3)	0.7682
II	8 (29.6)	11 (33.3)	0.7878
III	12 (44.5)	12 (36.4)	0.6011
IV	1 (3.7)	1 (3.0)	1.0
**Histological type** (%)			
Serous	22 (81.5)	19 (57.6)	0.0567
Clear	1 (3.7)	6 (18.2)	0.1161
Endometrioid	3 (11.1)	2 (6.1)	0.6494
Mucinous	0 (0)	5 (15.1)	0.0582
Others	1 (3.7)	1 (3.0)	1.0

Statistical analysis employed Chi-squared test in terms of stage and histological type.

## DISCUSSION

By high throughput functional siRNA screening, we identified Furin as a novel therapeutic target that shows a synthetic lethal interaction in c-Myc-driven ovarian cancer. Furthermore, we demonstrated that c-Myc cooperated with Furin to promote the proliferation of ovarian cancer cells, and that co-expression of c-Myc and Furin correlated with poor survival of ovarian cancer patients. Although c-Myc contributes to the genesis and progression of many human cancers, the association between c-Myc and Furin has not been known until now. To our knowledge, this is the first study to report that c-Myc cooperates with Furin in cell proliferation.

In our study, high throughput functional siRNA screening identified molecules associated with protein degradation, such as Furin, ADAMs, and CAPN (Supplementary Figure 1), as therapeutic targets. For example, ADAM15 is overexpressed in bladder, prostate and breast cancers and its substrate include EGFR ligands and TGF-β that are related to cancer cell proliferation [39]. These results suggest that protein degradation may be essential for proteins synthesized by deregulated c-Myc to perform their oncogenic functions, as we have indicated with Furin (Figure 4G).

We propose a model in which Notch1-mediated cell proliferation is involved in the synthetic lethal interaction between c-Myc and Furin (Figure 4G). Deregulated c-Myc increases the expression of pro-Notch1. It is first cleaved by Furin and subsequently cleaved by ADAM and γ-secretase. Released Notch1 intracellular domain translocates into the nucleus and activates target genes, which promotes cell proliferation. Furin inhibition suppresses the expression of mature Notch1 and subsequently cell proliferation, and leads to cell death. Notch1 directly upregulates c-Myc expression in leukemia, lymphoma, and breast cancer cells [40–42]. The Notch1-c-Myc regulatory loop may also be related to promoting cell proliferation in c-Myc-driven ovarian cancer.

Notch1 drives cell proliferation by inducing cell cycle regulators, such as cyclin D1, cyclin-dependent kinase 2, and c-Myc, and inhibiting cyclin-dependent kinase inhibitors [36]. Notch1 inhibition causes cell cycle arrest at G0/G1 in ovarian cancer cells [43] and glioma cancer cells [44], which is consistent with the result of cell cycle analysis upon inhibition of Furin in c-Myc-driven ovarian cancer (Figure 2B). This result supported that Notch1 was one of the key players in the synthetic lethal interaction between c-Myc and Furin.

Although it is controversial whether c-Myc directly regulates Notch1 expression [32, 45], Zeller *et al.* showed that Notch1 is one of the genes associated with c-Myc-binding sites identified by chromatin immunoprecipitation-paired end tag [33]. The database search revealed that the promoter region of Notch1 contains an E-box, a known c-Myc target sequence (Figure 4A). In addition, we found that Notch1 expression was upregulated by the c-Myc level in ovarian cancer cells (Figure 4C–4E). These results support the hypothesis that Notch1 is a target gene of c-Myc.

Notch1 is frequently expressed in ovarian cancers [35, 46]. Notch1 inhibition suppresses cell growth of ovarian cancer, indicating that Notch1 is a potential candidate for targeted therapy of ovarian cancer [35]. Although the library of 6,550 genes used in the high throughput siRNA screening included the Notch1 gene, it was not identified as a hit gene (Supplementary Figure 6). This result suggests that Notch1 cannot be a therapeutic target that shows a synthetic lethal interaction with c-Myc beyond Furin because in addition to Notch1, other substrates of Furin may be also involved in the synthetic lethal interaction between c-Myc and Furin.

The limitation of this study is that non-isogenic ovarian cancer cell lines, TOV112D and CAOV3, were used in high throughput siRNA screening. The cell system with same genetic background other than c-Myc such as Myc-estrogen receptor (Myc-ER) inducible system is ideal for the primary screening, but there was no suitable isogenic ovarian cancer cell pair. Instead, we confirmed the synthetic lethal interaction between c-Myc and Furin in several cell line pairs including HFF-p-Babe and HFF-c-Myc, with same genetic background other than c-Myc.

Furin is often overexpressed in many types of cancers, including head and neck, ovarian, breast, skin, colon, and lung cancers [29, 38, 47–50], and has been proposed as a potential therapeutic target. However, clinical or genetic characteristics to predict the efficacy of Furin inhibition have not been reported. In our study, Furin inhibition significantly suppressed the proliferation of high c-Myc cells compared with low c-Myc cells (Figure 1C–1G). High c-Myc may be a biomarker to predict the clinical efficacy of a Furin inhibitor, suggesting personalized medicine for Furin-targeted therapy.

Dec-RVKR-CMK was developed as the first potent peptidomimetic Furin inhibitor by incorporating a CMK [51]. Although no specificity for Furin was reported, this irreversible inhibitor was used to study the role of Furin in therapeutic effects [29, 52]. The *in vitro* inhibitory activity of Dec-RVKR-CMK has a dissociation constant of 1 nM. This concentration was not sufficient to inhibit the proliferation of ovarian cancer cells in a cell-based assay (Figure 1F). This large diversity may be due to low cell permeability. Although a wide variety of Furin inhibitors has been reported, including small molecules [53, 54], no clinical trials have been performed because of cytotoxic effects and instability. Further studies will be required for the development of a promising Furin inhibitor. In addition, an approach for Furin silencing, Vigil immunotherapy (GMCSF/bi-shRNA furin DNA engineered autologous tumor cell product) is expected in the future, as demonstrated in a phase I/II clinical trial for patients with advanced ovarian cancer [55, 56].

In summary, this study demonstrated a cooperative pathway of c-Myc and Furin in promoting cell proliferation of ovarian cancer. Furin may be a promising therapeutic target in c-Myc-driven ovarian cancer for precision medicine.

## MATERIALS AND METHODS

### Materials

Antibodies specific for Furin (sc20801) and c-Myc (sc40), and horseradish peroxidase-conjugated anti-mouse (sc2005) and -rabbit (sc2004) IgGs were purchased from Santa Cruz Biotechnology (Dallas, TX, USA). The anti-Notch1 antibody (#3608) was purchased from Cell Signaling Technology (Danvers, MA, USA). β-Actin (A5441) and V5-cy3 (V4014) antibodies were obtained from Sigma-Aldrich (St. Louis, MO, USA). Mouse monoclonal V5 antibody (R960-25), Lipofectamine RNAi Max (#13778150), Lipofectamine 2000 (#11668019), Lipofectamine 3000 (#L3000150), siRNA oligonucleotides directed against Furin (#1 s9987 and #2 s9988), c-Myc (#1 s9129 and #2 s9130), and notch1 (#1 s9634 and #2 s9633), and a negative control siRNA (#4390843) were from Thermo Fisher scientific (Waltham, MA, USA). Hoechst 33342 was from Dojindo (Japan). Furin inhibitor ALX-260-022 was from Enzo Life Sciences (Farmingdale, NY, USA). The CellTiter-Glo Luminescent Cell Viability Assay kit was from Promega (Madison, WI, USA). Human ovarian cancer cell lines (TOV112D, CAOV3, IGROV1, IGROVCP, DOV13, SKOV3, A2780, A2780CP, PE01, PE04, ES2, and TOV21G) were obtained from the American Type Culture Collection. OSE2 and OSE4 cell lines were kindly provided by Dr. Makoto Nitta (University of Kumamoto, Kumamoto, Japan). JHOC5, JHOC7, and JHOC8 human ovarian cancer cell lines were kindly provided by Dr. Katsutoshi Oda (University of Tokyo, Tokyo, Japan). HFFs transfected with retroviral vectors (pBabe-puro and pBabe-c-Myc-puro) have been described previously [57].

### Cell culture

IGROV and cisplatin-resistant IGROV-CP cells were cultured in RPMI-1640 supplemented with 10% fetal bovine serum (FBS). Other cell lines were cultured in Dulbecco’s modified Eagle’s medium (DMEM) supplemented with 10% FBS. Cells were maintained at <80% confluence under standard incubator conditions (humidified atmosphere with 5% CO_2_ at 37°C)

### High throughput siRNA screening

The siRNA library targeting 6,550 human genes with three individual siRNAs per gene was obtained from Sigma-Aldrich. Screening was carried out at the Quellos High Throughput Screening Core, Institute for Stem Cell and Regenerative Medicine, University of Washington School of Medicine (Seattle, WA, USA). TOV112D and CAOV3 cells were seeded in triplicate on 384-well plates at 1,000 cells per well in 50 µl medium (DMEM supplemented with 10% FBS and 10 mM HEPES). At 24 h after seeding, cells were transfected with 5 nM siRNAs using Lipofectamine RNAi Max. Semi-automated transfections were carried out using a Biomek FX. The plates were incubated at 37°C in a 5% CO_2_ incubator for 72 h. Cell viability was assessed using the CellTiter-Glo Luminescent Cell Viability Assay. Cell proliferation is described as the percentage of cell viability relative to the average value of three control siRNAs. Hit genes were determined by a volcano plot that showed the relationship the between fold change and statistical significance between TOV112D and CAOV3 cell viability.

### Small scale screening

Potential hit genes selected from high throughput screening and network analysis were validated by small scale screening. TOV112D and CAOV3 cells seeded in 96-well plates were transfected with control siRNA or siRNAs against individual genes. After transfection for 72 h, cell viability was measured using the CellTiter-Glo Luminescent Cell Viability Assay.

### Immunoblotting

Cells were washed three times with PBS containing 10 mM EDTA and then lysed using Laemmli sample buffer (Wako). Protein samples (5 or 10 μg) were subjected to SDS-polyacrylamide gel electrophoresis (4–20% gradient gels). Proteins were electrophoretically transferred to nitrocellulose membranes, blocked with PBS/0.1% Tween 20 (PBS-T) containing 5% nonfat dried milk, washed with PBS-T, and incubated with antibodies against Furin (1:1,000 dilution) in PBS-T containing 5% nonfat dried milk overnight. The anti-c-Myc antibody (1:200 dilutions) was diluted with PBS-T containing 2% bovine serum albumin. The membranes were washed three times with PBS-T and then incubated with a secondary antibody conjugated with horseradish peroxidase (1:5,000 dilutions) in PBS-T containing 5% nonfat dried milk for 1 h. Detection was performed using Supersignal West Dura Extended Duration Substrate (Thermo Fisher Scientific). Quantification of the chemiluminescent signals was performed with a digital imaging system (ChemiDoc, Bio-Rad)

### Cell viability assay

Cell viability was assessed with the CellTiter-Glo Luminescent Cell Viability Assay. Cells were seeded on 96-well plates at a density of 1–4 × 10^3^/well. After treatment with siRNA or Dec-RVKR-CMK, CellTiter-Glo reagent was added to each well, and then the plate contents were mixed on an orbital shaker. Luminescence was quantified on a standard luminometer.

### Cell cycle analysis

TOV112D cells (1 × 10^6^/100 mm dish) were trypsinized and fixed with ice-cold 70% ethanol overnight. Fixed cells were washed with PBS and stained with PI in the presence of RNase. Cellular fluorescence was quantitated using the FL3 channel of a flow cytometer (FACSCanto II, BD Bioscience). The cell cycle distribution was determined using FlowJo software.

### Annexin V/PI staining assay for apoptosis

The Dead Cell Apoptosis Kit (Life Technologies) was used to detect apoptotic cells. TOV112D cells (1 × 10^6^/100 mm dish) were trypsinized, washed with PBS, and stained with Alexa Fluor-488-conjugated annexin V and PI. Fluorescence was analyzed by the FACSCanto II flow cytometer

### Construction of c-Myc and Furin expression vectors

Human Furin cDNA was amplified by PCR using a set of primers containing a HindIII site (HindIII-start1F, aaggtaagcttatggagctgagg) and XbaI site (XbaI-nostopR, aaatctagagagggcgctctg) without a stop codon. c-Myc was amplified by PCR using a set of primers containing a HindIII site (HindIII-start1F, ttcgaagcttatggatttttttc) and XbaI site (XbaI-nostopR, aaatctagagagggcgctct) without a stop codon. Individual PCR products and pcDNA3.1/V5-His A were digested with HindIII/XbaI, and then the fragments were ligated with T4 DNA ligase (Takara, Japan). These vectors were amplified in *E. coli*.

### Transfection of siRNAs and plasmid vectors

For siRNA transfection, cells (2 × 10^5^/60 mm dish or 1 × 10^3^/96-well plate) were transfected with 5 nM siRNAs specific for Furin, c-Myc, or Notch1, or negative control siRNA using Lipofectamine RNAi Max transfection reagent. For transfection of plasmid vectors, cells (3 × 10^5^/35 mm dish or 3–4 × 10^3^/96-well plate) were transfected with plasmid vectors using Lipofectamine 2000. Lipofectamine 3000 was used for transfection of CAOV3 cells with vectors.

### Quantitative real-time PCR

RNAs were extracted using the RNeasy Mini Kit (Qiagen, Hilden, Germany) according to the manufacturer’s manual. After reverse transcription of the RNAs with SuperScript III (Thermo Fisher Scientific), quantitative real-time PCR was performed using the StepOne Plus Real-Time PCR System. TaqMan probes (Thermo Fisher Scientific) specific for c-Myc (Hs00153408_m1), Notch1 (Hs01062014_m1), Notch4 (Hs00965889_m1), TGF-β1 (Hs00998133_m1), TGF-β2 (Hs00234244_m1), TGF-β3 (Hs01086000_m1), and glyceraldehyde-3-phosphate dehydrogenase (GAPDH) (4333764F) were employed. GAPDH was used as an internal control.

### Human tissue samples

A total of 97 human ovarian cancer tissues ware obtained from the Surgical Pathology Archives at the Department of Obstetrics and Gynecology, Tohoku University Hospital (Sendai, Japan). Tissues were fixed in buffered formalin and embedded in paraffin. This study was approved by the Ethics Committee of the Tohoku University Graduate School of Medicine (Sendai, Japan).

### Immunohistochemistry

After deparaffinization, antigen retrieval was performed by heating in an autoclave at 121°C for 5 min in citrate buffer (pH 6.0). Samples were stained using the streptavidin-biotin amplification method and Histofine SAB-PO kit (Nichirei). An anti-Furin antibody (sc20801, Santa Cruz Biotechnology) was used as the primary antibody at a 1:500 dilution. Staining for c-Myc was performed by a Ventana automatic stainer (Roche, Basel-Stadt, Switzerland) according to the manufacturer’s protocol. An anti-c-Myc antibody (Y69, Roche) was used as the primary antibody. Immunohistochemical staining was evaluated and scored as follows: no or <10% positive cells was defined as “low expression” and ≥10% positive cells was defined as “high expression”. Three independent, blind observers evaluated immunostained section.

### Statistical analysis

Statistical analysis was performed using GraphPad Prism version 5. All statistical analysis employed the unpaired two-tailed *t*-test unless indicated otherwise. *P* < 0.05 was considered as statistically significant. Survival curves were constructed according to the Kaplan-Meier method and compared using the log-rank test.

## SUPPLEMENTARY MATERIALS FIGURES


