# Supplementary material for: Novel cooperative pathway of c-Myc and Furin, a pro-protein convertase, in cell proliferation as a therapeutic target in ovarian cancers
Source: Oncotarget. 2017 Dec 15;9(3):3483–96. doi: 10.18632/oncotarget.23322 (PMC5790477; doi:10.18632/oncotarget.23322)
Supplement: Supplementary file 1 [file oncotarget-09-3483-s001.pdf]

Novel cooperative pathway of c-Myc and Furin, a pro-protein convertase, in cell proliferation as a therapeutic target in ovarian cancers

SUPPLEMENTARY MATERIALS

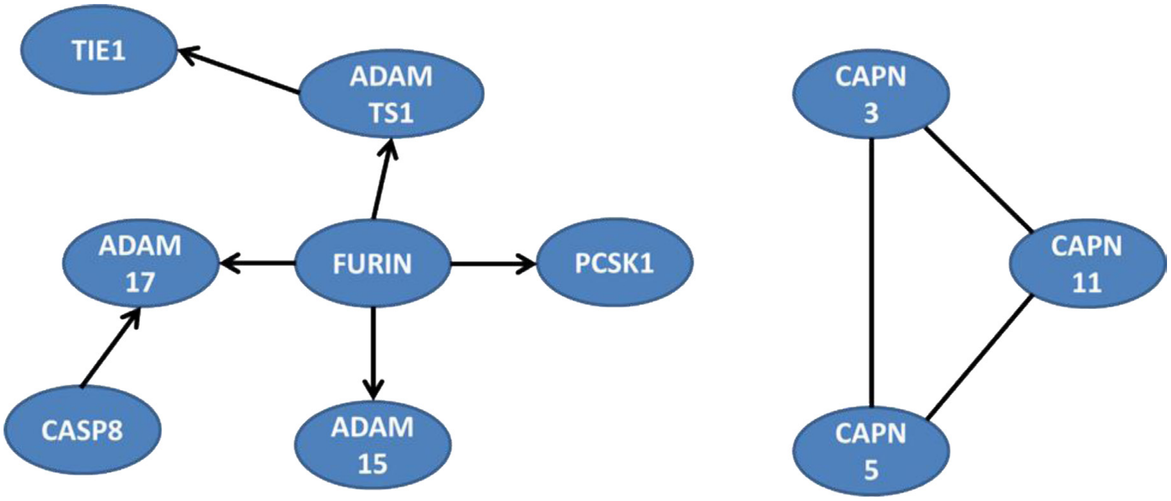

Supplementary Figure 1: Network analysis of the top 94 genes. Protein-protein interactions between the top 94 genes, which showed synthetic lethality with c-Myc, were analyzed by IPA, and two networks were detected.

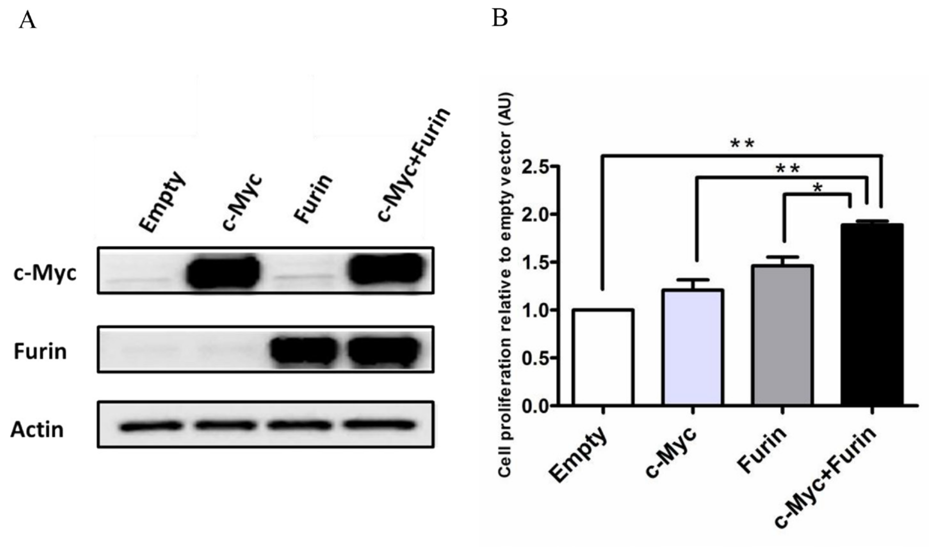

Supplementary Figure 2: c-Myc cooperates with Furin to promote cell proliferation. (**A**) CAOV3 cells (4 × 103/well) seeded on 96-well plates were transfected with empty, V5-tagged Furin, or c-Myc expression vectors (0.1 µg cDNA) at 24 h before harvesting. Extracted proteins were subjected to immunoblot analysis with antibodies specific for Furin or c-Myc. (**B**) Proliferation of CAOV3 cells was assessed at 72 h after transfection using CellTiter-Glo. Data shown were normalized to the empty vector group. Values are means ± SE. $^{*}p < 0.05$, $^{**}p < 0.01$.

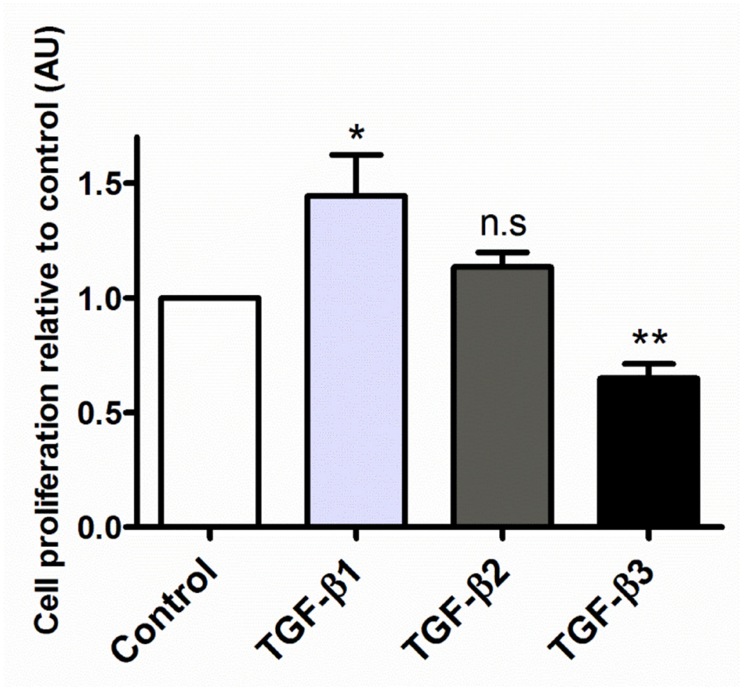

Supplementary Figure 3: Effects of TGF-β on proliferation of TOV112D cells. TOV112D cells (5 × 10^4/well) seeded on 6-well plates were incubated overnight. After 3 h of serum starvation with 1% FBS, cells were treated with recombinant TGF-β1, β2, or β3 (10 ng/ml) for 72 h, and then the cell number was determined. Data shown were normalized to the control. Values are means±SE. $^{*}p < 0.05$. $^{**}p < 0.01$. n.s: not significant.

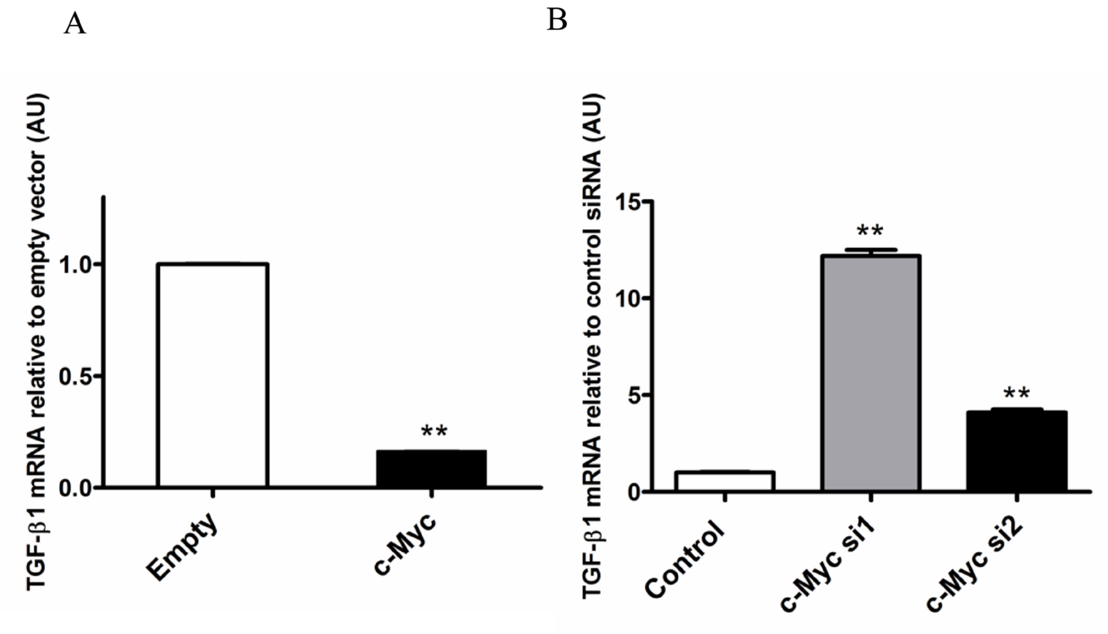

Supplementary Figure 4: Changes in TGF-β1 mRNA levels after c-Myc overexpression and knock down. (**A**) TOV112D cells were transfected with 1 μg empty or c-Myc expression vectors for 24 h. TGF-β1 mRNA levels were determined by quantitative real-time PCR. (**B**) TOV112D cells were transfected with 5 nM control or c-Myc siRNAs for 48 h. TGF-β1 mRNA levels were determined by quantitative real-time PCR. Data shown were normalized to empty vector or control siRNA groups. Values are means ± SE. $^{**}p < 0.01$.

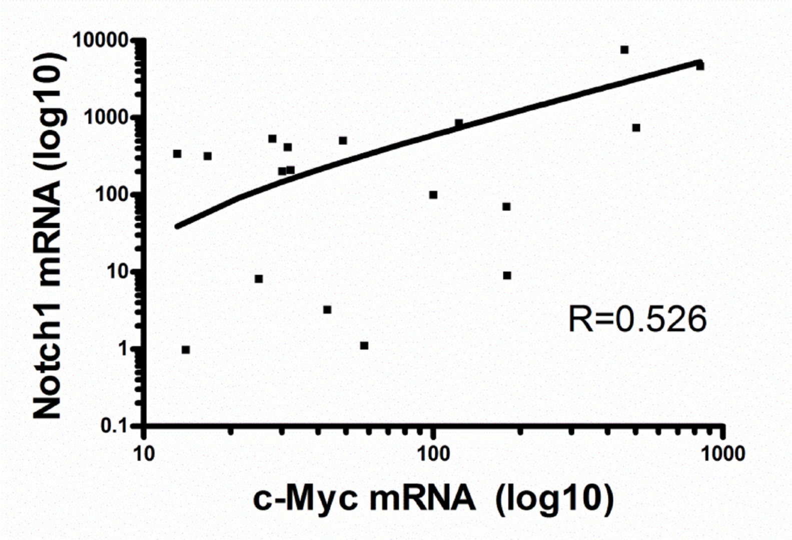

Supplementary Figure 5: Correlation of Notch1 and c-Myc mRNA expression levels in 18 cell lines. Notch1 and c-Myc mRNA expression levels in 18 cell lines, including 16 ovarian cancer cell lines, HFF-pBABE, and HFF-c-Myc, were determined by quantitative real-time PCR.

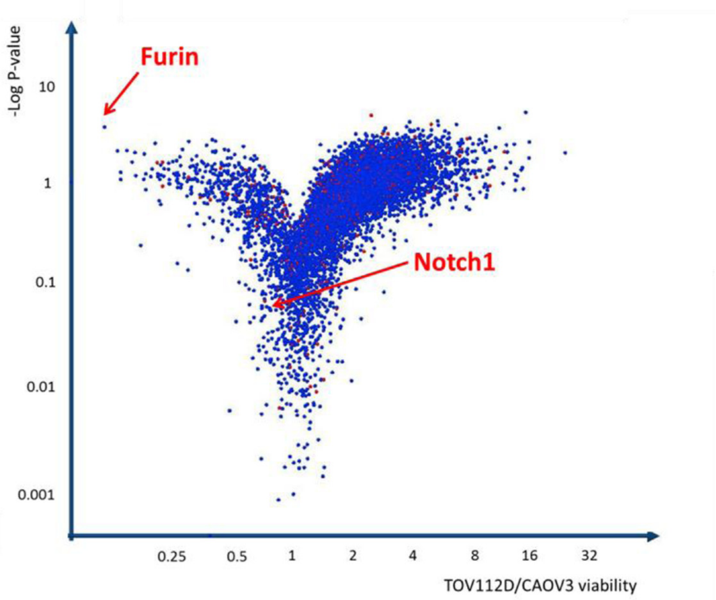

Supplementary Figure 6: Position of the Notch1 gene in a volcano plot of high throughput functional siRNA screening. The red arrow indicates Notch1 and the top hit gene, Furin.
